# Post-COVID-19 cavitary lung lesion due to *Aspergillus flavus* and *Enterobacter cloacae* in a patient suffering from COVID-19 pneumonia – a case report

**DOI:** 10.1099/acmi.0.000457

**Published:** 2023-06-22

**Authors:** Kamlesh Rajpal, Shailesh Kumar, Kumar Saurabh, Namrata Kumari, Rakesh Kumar, Randhir Kumar, Sweta Muni

**Affiliations:** ^1^​ Department of Microbiology, Indira Gandhi Institute of Medical Sciences, Patna, Bihar, India

**Keywords:** COVID-19, pulmonary cavitation, coinfection

## Abstract

The coronavirus disease 2019 (COVID-19) pandemic has manifested as a multifaceted paradigm but has primarily affected the respiratory system. Though a rare sequela after-COVID-19, we present a case of cavitary lung lesion in an adult patient, which manifested with common symptoms such as fever, cough and dyspnoea during the post-COVID-19 recovery period. *Aspergillus flavus* and *Enterobacter cloacae* were found to be the main causative organisms. Fungal and bacterial coinfection may be thought of in similar situations and appropriate treatment may be given to prevent further morbidity and mortality.

## Introduction

The coronavirus disease 2019 (COVID-19) pandemic has affected us globally and is posing a continuous challenge for clinicians in diagnosing and treating various post-COVID-19 complications. Pulmonary cavitation has been found to be a rare sequela in the post-COVID-19 recovery period [1, 2]. In general, it is presumed that the organisms causing chronic or subacute pulmonary infections (e.g. fungi and mycobacteria) are more frequently associated with cavities than the organisms causing acute pulmonary infections (e.g. *Streptococcus pneumoniae* and viruses) [3]. Pulmonary lesions caused by viral infection will be dissipated completely and absorbed gradually with time. The interstitial inflammation and fibrosis will be retained by a few severely infected patients [1].

We present a case of COVID-19 pneumonia, where multiple cavitary lesions developed in the lung due to *Aspergillus flavus* and *Enterobacter cloacae *, during the post-COVID-19 recovery phase.

## Case history

A 65 year-old hypertensive and non-diabetic male presented with fever, cough and dyspnoea for 3 days in the Department of Pulmonary Medicine. Physical examination showed the presence of bilateral rhonchi and crepts. RT-PCR assay for COVID-19 (using the Q-Line Molecular ER nCOV-19 RT-PCR kit) was found to be positive (*C*t-value 21) on the day of admission. Further, high-resolution computed tomography (HRCT) of lung showed peripheral ground glass opacity along a major fissure in bilateral lung parenchyma, focal areas of subpleural consolidation in the right middle, right lower and left lower lobes, subpleural band opacity in all the lobes (lower lobe>upper lobe) and vascular prominence in the bilateral lower lobe.

A diagnosis of moderate COVID-19 pneumonia with CO-RADS 4, CT severity score 15/25 was made. Among other laboratory tests, viral markers (HIV, HBsAg and HCV) were negative, haemoglobin was 11.4 g dl^−1^ (13.3–16.2 g dl^−1^), platelet count was 2.66 lac/cmm (1.65–4.15 lac/cmm), total erythrocyte count was 4.06 million/cmm (4.3–5.6 million/cmm) and total leucocyte count was 18910/cmm (4000–11000/cmm), with raised neutrophil and decreased lymphocyte count. Uric acid was 4.5 mg dl^−1^ (3.1–7.0 mg dl^−1^), serum sodium was 129 mmol l^−1^ (136–146 mmol l^−1^), serum potassium was 3.9 mmol l^−1^ (3.5–5.0 mmol l^−1^) and serum ionized calcium was 5.3 mg dl^−1^ (4.5–5.3 mg dl^−1^). Renal function tests were within normal limits, whereas in liver function tests, the SGPT level was 154 U l^−1^ (7–41 U l^−1^). C-Reactive protein (CRP) was 37.6 mg l^−1^ (0.2–3.0 mg l^−1^), the serum ferritin level was 1102.24 ng ml^−1^ (29–248 ng ml^−1^), the IL-6 level was 37.26 pg ml^−1^ (0–16.4 pg ml^−1^) and the plasma d-dimer level was 0.88 µg ml^−1^ (0.22–0.74 µg ml^−1^).

The patient was put on intravenous steroids (dexamethasone) with supplemental oxygen. Subcutaneous enoxaparin was started along with IV piperacillin–tazobactam and anti-pyretics. During further intensive care unit (ICU) stay, the clinical condition of patient deteriorated from the third day, with laboratory parameters showing raised total leucocyte count (24450/cmm), raised neutrophil count (89%), raised CRP (46 mg l^−1^), raised serum ferritin (1112.89 ng ml^−1^), raised LDH (310.3 U l^−1^), raised SGPT (232 IU l^−1^) and raised SGOT (56 IU l^−1^). Random blood sugar was found to be raised (310 mg dl^−1^), which might have been due to the ongoing IV steroid administration.

With the onset of sepsis and suspicion of COVID-associated mucormycosis, blood culture and sputum culture were ordered for detection of any possible bacterial or fungal aetiology. The blood culture was negative after 5 days of aerobic incubation by the BacT/ALERT automated blood culture system. Sputum culture was also not helpful as only mixed oral commensals were obtained in culture. Ziehl–Neelsen staining of the sputum sample and its culture using the mycobacteria growth indicator tube (MGIT) technique was negative for *Mycobacterium tuberculosis* complex (MTC) and non-tuberculous mycobacteria (NTM). The fungal culture of sputum performed on Sabouraud dextrose agar (SDA) medium showed growth of *A. flavus* but no anti-fungals were started at this juncture, as the finding was considered to be a laboratory contaminant by the treating physicians.

**Fig. 1.**
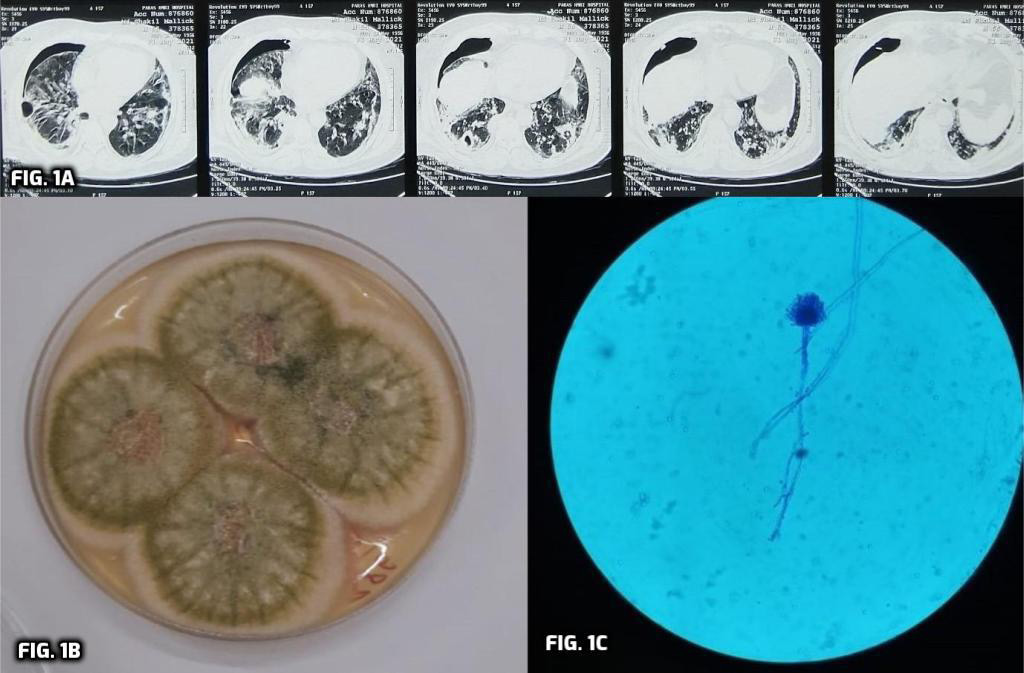
(a) Multiple thick-walled peripheral cavities in bilateral lung parenchyma, diffuse patchy consolidation in bilateral lower lung lobes and hydropneumothorax in the right pleural space (chest HRCT image). (b) *Aspergillus flavus*. The obverse side of the culture plate shows a velvety, yellow to green colony. (c) The LPCB mount showing biseriate phialides covering the entire vesicle.

Because of the deteriorating clinical condition, the patient was switched to meropenem, clindamycin and moxifloxacin, which continued for 2 weeks, with some improvement in the clinical condition. After 2 weeks, the patient again developed cough and severe dyspnoea. Repeat HRCT of the chest showed multiple thick-walled peripheral cavities in bilateral lung parenchyma, raising the possibility of bacterial and/or superadded fungal infection. Further, there was diffuse patchy consolidation in bilateral lower lung lobes and hydropneumothorax in the right pleural space (Fig. 1a).

A repeat sputum culture was performed. This time, the sputum culture, performed on blood and MacConkey agar, revealed pure growth of *E. cloacae,* which was further confirmed by the VITEK-2 automated identification and sensitivity testing system. The organism was sensitive to amikacin, gentamicin, imipenem, meropenem, cefotaxime, cefepime, aztreonam, piperacillin–tazobactam, cotrimoxazole, ciprofloxacin, levofloxacin and tetracycline, whereas it was resistant to cefazolin, cefoxitin, ampicillin and co-amoxiclav. Accordingly, levofloxacin and cefepime were started. The sputum sample was also sent for GeneXpert analysis for *Mycobacterium tuberculosis* (MTB) and repeat fungal culture. GeneXpert analysis was negative for MTB but fungal culture on SDA medium containing chloramphenicol, incubated at 25 °C, revealed the growth of *A. flavus* on the fourth day of incubation, with a velvety yellow to green colony having a granular texture on the obverse side (Fig. 1b), and a cream-coloured colony on the reverse side. The culture isolate was confirmed phenotypically using the slide culture technique and the lactophenol cotton blue (LPCB) mount. On the LPCB wet mount, the presence of uniform, septate fungal hyphae with parallel walls and branching at acute angles was observed. Rough, pitted and spiny conidiophores with a biseriate arrangement of phialides covering the upper two-thirds or the entire vesicle were observed. (Fig. 1c).

The identification of *A. flavus* was further confirmed using a matrix-assisted laser desorption/ionization time-of-flight mass spectrometry (MALDI-TOF MS) instrument.

As far as serological tests are concerned, it was only possible to determine the serum galactomannan level by enzyme immunoassay, and this was found to be negative.

Based on the above findings, voriconazole 200 mg every 12 h orally was immediately added to the ongoing therapy. After 7 days of meticulous treatment, the patient showed signs of clinical improvement and was subsequently discharged with advice to continue the same dose of antibiotic and anti-fungal treatment for 2 and 5 more weeks, respectively, with further follow-up.

## Discussion

Cavitary lesions in the lung due to COVID-19 pneumonia is an uncommon finding [1]. The exact mechanism of cavitation in COVID-19 pneumonia is unknown but the available literature suggests that it may be related to diffuse alveolar damage, intra-alveolar haemorrhage and necrosis of parenchymal cells [4, 5]. A few cases of cavitation in the lung have also been reported in COVID-19 pneumonia patients, despite ongoing steroid therapy [4]. As compared to other parts of the world, many cases of COVID-19 pneumonia in India were found to be complicated with mucormycosis [6], compelling clinicians to have a suspicious eye for the same. In this suspected case of mucormycosis, the sputum sample was found to be positive for *A. flavus* but negative for any other zygomycetes. The host characteristics and clinical manifestations overlap considerably between zygomycosis and invasive pulmonary aspergillosis [3], which must always be kept in mind while making a diagnosis.

Interestingly, the serum galactomannan level was negative in this case, which shows that depending on this marker totally for diagnosis of *Aspergillus* sp. may be misleading. The low sensitivity and low positive predictive value of serum galactomannan are well documented [7, 8]. The detection of galactomannan in specimens other than serum specimens may provide additional evidence for invasive aspergillosis via a noninvasive method and may help to exclude false-positive or false-negative test results obtained using serum samples [9], which could not be done in this case. At the same time, other serological tests such as β-1-3-d-glucan assay, other lateral flow assay (LFA)-based point-of-care tests or IgG antibody tests to detect *Aspergillus* antibodies, including Immy, Serion/Virion, Bioenche, Bio-Rad, Thermo Fisher, Elitech, Microgen, Meridian LD Bio and Siemens, may be performed [9].

Along with *A. flavus,* the bacterial culture of sputum also revealed the presence of *E. cloacae *. The pulmonary cavities after bacterial infections may be caused by one of two mechanisms. First, entry of organisms in the respiratory cavity via the oropharynx/upper airway, bypassing host defences, and causing either a lung abscess or necrotizing pneumonia. Alternatively, entry through the bloodstream is also possible, often in association with platelets and fibrin as septic pulmonary emboli. In this case, as the patient was immunocompromised, he might have acquired *E. cloacae* infection through the respiratory tract during his ICU stay. *Klebsiella pneumoniae, Prevotella*, *Fusobacterium* and streptococci (particularly the *Streptococcus milleri* group) have frequently been isolated from lung abscesses, resulting in cavitary lesions [10–12]. *Staphylococcus aureus* has also been found to cause cavitary pneumonia [13]. Few cases of cavitary pneumonia have been reported with *Streptococcus pneumoniae* [14] *or Haemophilus influenzae* [15]. There are very few reports with *E. cloacae* as the cause of necrotizing pneumonia in the case of ICU patients on mechanical ventilation [16]. *E. cloacae* was also isolated from the sample in this case, which further signifies the role of this bacteria in causing post-COVID-19 cavitary lung lesion.

Studies have been performed to establish the hypothesis that SARS-CoV-2 may co-exist in biofilms with other micro-organisms, which needs more evidence and clinical observation [17]. Most biofilms are surface-associated with biotic (e.g. epithelial or dental surfaces) or abiotic surfaces, whereas others can be untethered microbial aggregates that colonize compromised tissue compartments (e.g. sputum within the lumen of cystic fibrosis airways) [18]. In this case also, the possibility of biofilm formation cannot be ruled out with bacteria such as *E. cloacae* growing in it, showing no response to prior antibiotic (meropenem) therapy. Overall, *A. flavus* seems to have been more responsible for the patient’s condition, but co-infection with *E. cloacae* must have added to the increased morbidity. *In vitro* synergy between β-lactams and fluoroquinolones against Gram-negative organisms has ranged from 17–82% and has been shown in many studies [19, 20]. Seeing the severity of infection in this case, the combination of levofloxacin and cefepime was used, which definitely helped the patient. Cefepime is a fourth-generation cephalosporin with a better spectrum of activity and has also been shown to have synergistic action with levofloxacin in the past [21]. A combination of antimicrobials is routinely used for the treatment of biofilms, with the aim of preventing or delaying the onset of resistance [22]. Combination therapy with levofloxacin and cefepime has definitely worked in this case as compared to meropenem therapy alone. *M. tuberculosis *, though most commonly associated with cavitary pulmonary disease in developing countries [23], was not detected in this case.

## Conclusion

*Aspergillus flavus* may cause cavitary lung lesion in post-COVID-19 pneumonia patients and *Enterobacter cloacae* can add to the morbidity. Fungal and bacterial co-infection should be suspected and taken care of, while treating post-COVID-19 pneumonia patients. Even after broad-spectrum antibiotic therapy, certain bacteria (* E. cloacae* in this case) may become refractory due to possible biofilm formation. A combination of antimicrobials such as fluoroquinolones and beta lactams may play an effective role in such cases. Any initial fungal growth obtained from a patient’s sample must be thought of as a possible aetiological agent and correlated clinically, before declaring it to be a laboratory contaminant. The serum galactomannan level may be negative in cases of *Aspergillus* infection in post-COVID-19 patients. Therefore, we recommend detection of galactomannan in specimens other than serum specimens, which may provide additional evidence for invasive aspergillosis via a noninvasive method and may help to reconfirm any false-positive or false-negative test results obtained using serum samples. *Mycobacterium tuberculosis* may be absent in cases of post-COVID-19 cavitary lung lesion.
